# Stress and the Immune System: Insights From Psychoneuroimmunology

**DOI:** 10.33549/physiolres.935804

**Published:** 2026-06-01

**Authors:** Zuzana OZANIAK STRIZOVA

**Affiliations:** 1 Department of Immunology, Second Faculty of Medicine, Charles University and Motol University Hospital, Prague, Czech Republic

**Keywords:** Fight and flight, Cortisol, T cells, Antibodies, PTSD

## Abstract

Stress profoundly impacts the immune system, functioning as both an enhancer and suppressor of immune responses. The ultimate outcome depends on a combination of individual characteristics, genetic, epigenetic, and environmental factors, and the nature of the stressor. Stress hormones influence leukocyte trafficking, activity, cytokine production, and the overall immune function, leading to immune dysregulation. This review offers a comprehensive perspective on the physiology of the stress response and its effects on immunity, particularly stress-induced alterations in immune cell function. It explores how stress contributes to immunodeficiencies, allergies, and autoimmune diseases, thereby impacting vaccine efficacy, increasing susceptibility to infections, and promoting cancer development. Stress is implicated in the onset and exacerbation of specific autoimmune disorders and contributes to allergic conditions, intensifying allergic responses in both adults and children, particularly in the context of parenting stress and domestic violence. This review also investigates immune changes associated with specific stressors, including bereavement, intimate partner violence, childhood maltreatment, caregiving stress, and post-traumatic stress disorder. This work addresses the multifaceted dimensions of psychoneuroimmunology, shedding light on the significant health consequences of stress-induced immune dysfunction, and emphasizes the need for integrative therapeutic approaches.

## Introduction

Stress is a widely recognized risk factor that significantly affects human health [1]. To date, it has been acknowledged that stress is a complex phenomenon consisting of a sequence of events initiated by a stimulus, also known as a stressor. The stressor, which may be physical or psychological, elicits a perceptual response in the brain and subsequently triggers a cascade of physiological reactions [1]. This stress response is associated with the activation and release of neurotransmitters and hormones, facilitating the initiation of the “fight or flight” adaptive mechanism [2]. The “fight or flight” model was initially introduced in the 1920s by American physiologist Walter Bradford Cannon [3]. However, the concept was expanded upon in later years to encompass additional physiological and behavioral responses known as the “freeze” and “fawn” [4]. These adaptive processes have been demonstrated to be effective in minimizing the potential for injury, thereby reducing the risk associated with threatening circumstances.

Freezing, also known as tonic immobility, can be triggered not only by the physical presence of a predator but also by cues such as the scent of the predator or direct eye contact [4]. In humans, tonic immobility has been documented in sexual assault victims as an involuntary state of motor and psychological inhibition during a traumatic event, temporarily preventing movement, vocalization, or resistance [5].

Available data regarding the fawn stress response, sometimes referred to as appeasement, remain relatively limited. The fawn stress response is marked by a sincere effort to please the predator and avoid any possible conflicts or confrontations [6,7]. Although the fawn response has been mostly demonstrated in dogs, in humans, fawning has been observed as a pattern of codependent behavior employed to mitigate harm within abusive dynamics involving caregivers or intimate partners.

Naturally, humans exhibit wide variations in their perception and handling of stress. Resilience to stress is believed to arise from a combination of genetic predispositions and environmental factors. These factors contribute to an individual’s ability to withstand and cope with stress more effectively [8].

Research has provided evidence that stress has a multifaceted impact on the immune system. It has the potential to either enhance or inhibit immune function, thereby contributing to the development of various immune-related disorders. Stress can also disrupt sleep patterns, alter eating habits, and lead to unhealthy coping mechanisms, all of which can further compromise the immune system [9].

The intensity and duration of stress play major roles in determining the extent to which immune functions are affected. Acute stress typically manifests for short durations ranging from minutes to hours, and rarely extends beyond a few days. Conversely, chronic stress is prolonged stress that persists for several hours a day, spanning weeks or even months [10]. The intensity of stress can be quantified by assessing the biological responses triggered within an individual. Over the years, researchers have relied on different indicators to measure stress levels. These indicators include assessing stress hormone levels, observing changes in neurotransmitter activity, monitoring alterations in heart rate and blood pressure, and examining modifications in glucose metabolism.

Understanding the interplay between stress and physiological responses is crucial for understanding the immunological consequences of prolonged exposure to stress.

## Complex interactions between the nervous, endocrine, and immune systems

The nervous, endocrine, and immune systems are functionally integrated and interact closely to maintain homeostasis, coordinate host defense, and influence both the pathogenesis and treatment of disease.

Communication between the immune system and the brain is bidirectional. Immune cells and their cytokines can affect neural activity through several mechanisms, including stimulation of peripheral afferent nerves, transport across the blood-brain barrier, and, under conditions of increased barrier permeability, direct entry of immune cells into the central nervous system. In addition, the brain itself produces cytokines such as IL-1, IL-6, and IFN-α [11]. During pain, cytokines released by microglia and macrophages, including IL-1β, TNF-α, and IL-6, enhance nociceptor sensitivity and contribute to both peripheral and central sensitization [12].

Peripherally produced cytokines can also influence brain function either by crossing the blood-brain barrier or indirectly *via* neural pathways such as the vagus nerve. Pro-inflammatory cytokines including IL-1β and TNF-α act on hypothalamic thermoregulatory centers to induce fever and also affect higher brain regions involved in appetite, energy balance, motivation, and mood, thereby contributing to sickness behavior [13].

Signaling also occurs in the opposite direction. The nervous system, through neurotransmitters such as adrenaline and noradrenaline, and the endocrine system, through hormones including glucocorticoids, estrogens, growth hormone, and thyroid hormones, regulate immune-cell proliferation, differentiation, and effector function. Through these pathways, neuroendocrine signals modulate the magnitude and quality of immune responses, including T and B cell activity, cytokine production, and inflammatory processes [14].

In addition to β-adrenergic signaling, endocrine control of glucose availability, uptake, and metabolism is essential for immune function. Because immune responses depend on cellular proliferation and clonal expansion, activated immune cells have high metabolic demands. IL-1 has been shown to support immune energetics by stimulating glucose uptake in lymphoid tissue [15]. More broadly, immune activation is accompanied by metabolic reprogramming that supports proliferation, cytokine synthesis, and effector activity.

A key feature of this reprogramming is increased expression of the glucose transporter GLUT1 and a shift toward aerobic glycolysis (the Warburg effect), in which glucose is converted to lactate despite the presence of oxygen [16]. Although less efficient in ATP yield, this pathway rapidly generates metabolic intermediates required for biosynthesis. This metabolic shift is particularly characteristic of pro-inflammatory immune cells, including effector T cells (Th1, Th17) and M1 macrophages. In contrast, regulatory T cells (Treg) and M2 macrophages rely more heavily on oxidative metabolism and fatty-acid β-oxidation [17].

Activated CD4^+^ and CD8^+^ T cells markedly upregulate GLUT1 to increase glucose uptake, and M1-polarized macrophages similarly enhance GLUT1 expression to support cytokine production and reactive oxygen species generation. These processes are regulated by signaling pathways involving transcription factors such as HIF-1α and c-Myc, which respond to inflammatory and hypoxic cues [18].

## Physiology of stress response

**Fig. 1:**
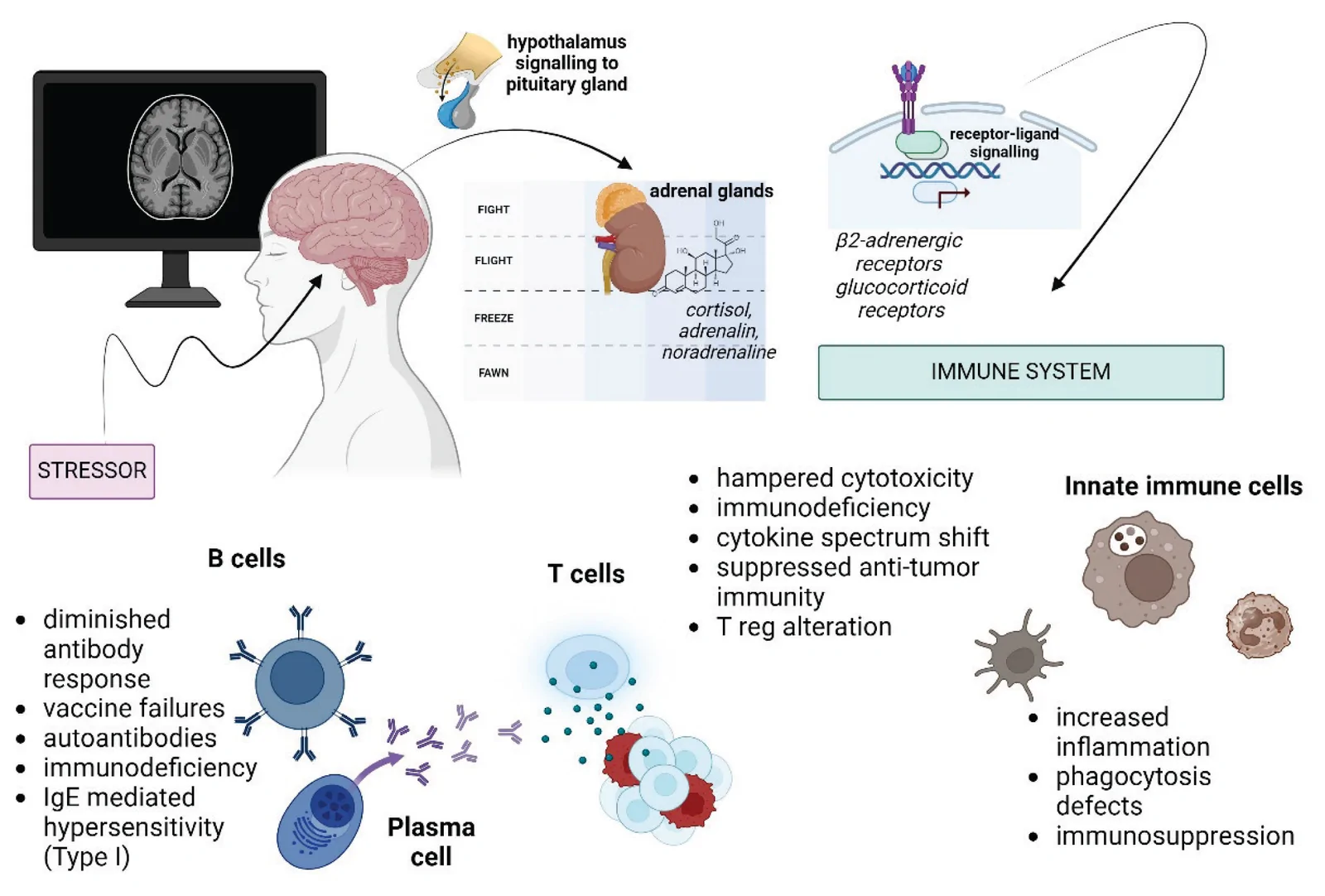
The interplay between psychological stressors and the immune system. Stressors activate the hypothalamic-pituitary-adrenal (HPA) axis, signaling the pituitary gland to stimulate the adrenal glands to release stress hormones, including cortisol, adrenaline, and noradrenaline. These hormones act through the β2-adrenergic and glucocorticoid receptors, modulating multiple immune functions. Created at https://BioRender.com.

The stress response follows a sequential pattern. It begins with the brain perceiving the stressor. After the brain senses a stressor, it sends a signal to the hypothalamus. In response, the hypothalamus activates the sympathetic nervous system, prompting adrenal glands to release adrenaline (Fig. 1) [19]. The released adrenaline circulates through the bloodstream, affecting various organs and tissues in the body and initiating a series of biological processes that result in an energy boost. Adrenaline exerts its physiological effects by increasing plasma glucose through stimulation of glycogenolysis and gluconeogenesis, while simultaneously promoting lipolysis, ketogenesis, and thermogenesis [20].

Simultaneously, the hypothalamus also activates the hypothalamic-pituitary-adrenal (HPA) axis. The hypothalamus releases corticotropin-releasing hormone (CRH), which stimulates the pituitary gland to release adrenocorticotropic hormone (ACTH) [21]. ACTH circulates through the bloodstream and prompts adrenal glands to release cortisol, a glucocorticoid stress hormone. Cortisol, in turn, suppresses non-essential bodily functions such as digestion and ovulation, focusing on the body’s resources in response to the stressor. It is noteworthy that although appetite tends to be suppressed during acute stress, chronic stress often stimulates the desire and consumption of high-fat and energy-rich foods [21].

Cortisol also plays a role in regulating glucose metabolism and promoting the availability of glucose for energy. Together with adrenaline, cortisol ensures increased heart rate, blood pressure, heightened sensory perception, and enhanced muscle strength, preparing for the fight-or-flight stress response [19]. In addition to stress hormones, neurotransmitters, such as serotonin, dopamine, noradrenaline, and gamma-aminobutyric acid (GABA), play a significant role in various aspects of the stress response [22].

The HPA axis is regulated by a mechanism known as negative feedback. This mechanism involves the inhibition of CRH in the hypothalamus and ACTH in the pituitary gland, which subsequently regulate the release of cortisol. However, in chronic stress, this negative feedback system becomes disrupted, and prolonged exposure to high cortisol negatively impacts the cardiometabolic and immune systems, as well as cognitive and mental functions. Additionally, the responsiveness of glucocorticoid receptors, through which cortisol exerts its effects, decreases during chronic stress and may even undergo downregulation [19,21].

## Acute stress and immune cell dynamics

When exposure to a stressor is brief and of low-to-moderate intensity, leading to transient activation of the sympathetic nervous system and HPA axis without chronic glucocorticoid elevation, it can enhance immune function [9]. Acute stress induces rapid redistribution of immune cells, a well-documented response observed across species and considered an adaptive mechanism preparing the organism for potential injury or infection. This leukocyte redistribution is primarily mediated by glucocorticoids and catecholamines, which drive swift changes in immune cell trafficking [23].

While glucocorticoids, such as cortisol, in cases of acute stress increase mobilization of neutrophils, monocytes, and lymphocytes from the bone marrow into the bloodstream, catecholamine hormones, including noradrenaline and adrenaline, modulate leukocyte trafficking by altering the expression of adhesion molecules on the surface of immune cells. This mechanism allows leukocytes to easily migrate to injured or endangered tissues and adhere to this specific site [23]. Through the modulation of leukocyte distribution, catecholamines effectively guide immune cells towards areas where their presence is required during stress-related events.

In a recent study by Poller *et al*., the authors exposed mice to different acute stressors and observed a decrease in the number of circulating monocytes and lymphocytes and an increase in the number of neutrophils. Interestingly, subjecting mice to only 30 s of restraint stress was sufficient to cause a significant increase in neutrophil counts; however, this increase was observed with a one hour delay. However, it took at least four hours of continuous restraint to reach maximum levels [24].

From a practical perspective, acute stress amplifies the timing and extent of leukocyte infiltration at the site of immune activation or surgical intervention. In another study, immune cell infiltration was quantified in mice at a surgical site following implantation of a foreign material [25]. Mice that experienced acute stress before having a foreign material implanted under their skin showed significantly higher levels of neutrophils, macrophages, natural killer (NK) cells, and T cells infiltrating the area than non-stressed mice. The authors also demonstrated that acute stress increases the trafficking of all leukocyte subpopulations to a site of immune activation. However, tissue damage, antigens, or pathogen-driven chemoattractants can further dictate the recruitment of specific subpopulations.

In humans, the acute stress response frequently elicits biphasic alterations in the immune cell counts. Initially, catecholamines cause a rapid increase in granulocytes and natural killer (NK) cells [26]. During this stage, granulocytes and NK cells exit their respective organs and enter the bloodstream, resulting in an observed increase in leukocyte levels in the laboratory tests. In the next stage, the HPA axis elicits the secretion of glucocorticoid hormones, and leukocytes migrate from the bloodstream and strategically position themselves at various immune-primed sites, such as the skin, lung, gastrointestinal, and urinary-genital tracts, mucosal surfaces, and lymph nodes. At this stage, the redistribution of leukocytes causes a decrease in the circulating white blood cells [26].

## Immunological consequences of chronic stress

The chronicity of stress usually arises when the underlying stressor cannot be resolved and the stress appears daily for several hours over a span of weeks or months [10]. Extensive evidence suggests that prolonged exposure to stressors can disrupt the delicate balance of the immune system, resulting in compromised or impaired immune response. This dysregulation may manifest as altered cytokine production, impaired antibody response, or reduced activity of immune cells, ultimately affecting the body’s ability to defend against pathogens and maintain optimal immune health [27].

Prolonged cortisol exposure during chronic stress induces glucocorticoid receptor desensitization through downregulation and reduced sensitivity, impairing hypothalamic-pituitary-adrenal (HPA) axis negative feedback and promoting sustained hypercortisolism associated with obesity, diabetes, and cardiometabolic disease [21].

While prolonged activation of the HPA axis due to chronic stress induces a notable reduction in blood leukocyte counts, it is important to mention that the response of ACTH to chronic stress can vary among individuals and can be influenced by factors such as genetic predisposition, previous stress exposure, and environmental factors [27].

It is noteworthy that the complex effects of stress on immune cells may not be solely attributable to increased signaling of stress hormones. There is also evidence suggesting that trauma-induced immune alterations can affect the responsiveness of immune cells to protective or regulatory signals. For instance, childhood maltreatment has been shown to be significantly associated with reduced expression of the oxytocin receptor in peripheral mononuclear cells. Furthermore, a negative association has been observed between oxytocin receptor protein expression and the severity of maltreatment exposure, indicating long-term alterations, particularly affecting oxytocin receptor expression in immune cells. Consequently, physiological immune processes, such as the release of anti-inflammatory cytokines, may be impaired following exposure to childhood maltreatment, potentially due to diminished oxytocin receptor expression. This mechanism may contribute to the development of a pro-inflammatory phenotype in adulthood associated with early-life adversity [28].

Understanding the impact of chronic stress on immune function is crucial to elucidate the complex interplay between psychological states and physiological well-being.

### β-adrenergic receptor signaling

β-adrenergic receptors (β-ARs) are G protein-coupled receptors within the sympathetic nervous system that bind the catecholamines epinephrine and norepinephrine, thereby mediating the classical “fight-or-flight” response. These receptors are subdivided into three subtypes, β_1_-, β_2_-, and β_3_-adrenergic receptors [29].

The β_1_-adrenergic receptor is predominantly expressed in the heart and brain, where it regulates cardiac output and central nervous system activity. In contrast, the β_2_-adrenergic receptor has a broader tissue distribution and is widely expressed in vascular, pulmonary, and immune cells. The β_3_-adrenergic receptor is primarily found in adipose tissue, where it plays a key role in metabolic regulation [30].

Within the central nervous system, β-adrenergic receptors facilitate sympathetic activation through noradrenergic projections originating from the locus coeruleus, which innervate key brain regions including the amygdala, prefrontal cortex, hippocampus, hypothalamus, and thalamus. Chronic psychosocial stress leads to sustained elevation of endogenous catecholamines. As an adaptive mechanism, this results in rapid β-AR internalization and functional desensitization, preventing excessive receptor stimulation [31].

In the immune system, adrenergic signaling exerts diverse and cell type-specific effects. Depending on the immune subset, β-adrenergic receptor activation can regulate processes ranging from cellular trafficking and migration to cytokine production and effector function.

Table 1 shows β_1_-adrenergic receptors on major immune subtypes. classic leukocyte β-adrenergic binding hierarchy plus the strong functional evidence for β_2_-driven mobilization/suppression, NK cells and monocytes, followed by CD8 cells might be the most affected immune cells by stress [32–35].

### Glucocorticoid receptor signaling

Glucocorticoid receptor signaling is functionally diverse and largely determined by the expression of multiple receptor isoforms [36]. The canonical isoform, GRα, binds glucocorticoids and mediates their classical anti-inflammatory and immunosuppressive effects. In contrast, GRβ does not bind glucocorticoids and acts as a dominant negative inhibitor of GRα. GRβ, thus, also contributes to glucocorticoid resistance [37].

Glucocorticoid receptors are widely expressed across immune cells. Activation of GRα, suppresses pro-inflammatory cytokine production (e.g., TNF, IL-1β, IL-6), promotes anti-inflammatory mediators such as IL-10, induces apoptosis in selected immune populations, and inhibits antigen presentation and co-stimulatory signaling. These effects are cell type-specific. In T cells, GR limits activation and favors regulatory T cell differentiation. In B cells, glucocorticoids reduce antibody production and induce apoptosis. In monocytes and macrophages, glucocorticoids suppress inflammatory responses and promote an anti-inflammatory phenotype, while in dendritic cells, they inhibit maturation and T cell priming. Lastly, in neutrophils, GR signaling though GRα, prolong survival while dampening inflammatory activity [37,38].

The balance between GRα and GRβ is a key determinant of immune responsiveness. High GRα activity supports effective anti-inflammatory signaling, whereas increased GRβ expression reduces glucocorticoid sensitivity and is associated with chronic inflammation, autoimmunity, and severe infection [39].

### Neuroimmune modulation of T cells through β_2_-adrenergic receptors

T cells are central to adaptive immunity and antitumor defense, recognizing antigens *via* the T cell receptor presented on major histocompatibility complex molecules. Stress disrupts T cell responses by reducing cell numbers, impairing proliferation, altering cytokine production, and promoting inflammatory dysregulation [40].

T cell responses can be modulated through the expression of β_2_-adrenergic receptors on T cells [41]. β_2_-adrenergic receptors are G protein-coupled receptors that initiate signaling cascades, resulting in increased levels of cyclic adenosine monophosphate (cAMP) and activation of protein kinase A. Activation of these receptors triggers many physiological effects, including smooth muscle relaxation, bronchodilation, increased heart rate, and increased blood flow to the skeletal muscles. Adrenergic receptors have been found to play a significant role in both CD4^+^ and CD8^+^ T cells [41].

During stress, several scenarios may occur upon the binding of stress hormones to β_2_-adrenergic receptors on T cells. First, the activation of these receptors was shown to cause a shift in cytokine production, leading to the promotion of anti-inflammatory cytokines, such as IL-10, and the suppression of inflammatory cytokines, such as tumor necrosis factor-alpha (TNF-α) and interferon-gamma (IFN-γ). Another important mechanism by which stress suppresses T cell function is modulation of T cell trafficking. The activation of β_2_-adrenergic receptors can disrupt the appropriate trafficking of T cells, thus causing their absence in the expected locations, such as infection sites or cancer tissue [41]. In particular, β_2_-adrenergic signaling in T cells alters the way these cells respond to chemokine cues. For example, activation of these receptors enhances CCR7/CXCR4 signaling, which diminishes the exit of T cells from lymphoid tissues and promotes their retention within lymph nodes. As a result, fewer T cells reach peripheral tissues [42].

In CD8^+^ T cells, memory phenotypes have been observed to exhibit higher expression levels of β_2_-adrenergic receptors than naïve T cells, which suggests that β_2_-adrenergic receptors may contribute to the regulation of memory T cell functions [43].

This aligns with numerous observations in vaccinated individuals, demonstrating that stress can impair the immunogenicity of antimicrobial vaccines, such as influenza vaccines or the SARS-CoV-2 vaccine [44]. Conversely, adrenergic receptor blockade enhances the capacity of CD8^+^ T cells to generate robust antiviral immune responses against influenza [45].

Bucsek *et al*. demonstrated that suppressing adrenergic signaling also reduces PD-1 levels in CD8^+^ T cells, potentially augmenting the effectiveness of checkpoint inhibition therapy [46]. Interestingly, another checkpoint receptor, cytotoxic T-lymphocyte-associated protein 4 (CTLA-4), has been shown to crosstalk with β_2_-adrenergic receptors. Furthermore, these receptors affect antigen cross-presentation by dendritic cells, thereby influencing T cell signaling [47].

In CD4^+^ T cells, β_2_-adrenergic receptors have been shown to play a specific role in Th1 responses. The activation of these receptors hampers the production of IFN-γ, a key cytokine involved in cellular immune responses. Consequently, this modulation impacts specific cellular responses mediated by CD4^+^ T cells and further affects the activation of macrophages [48]. Furthermore, the engagement of β-adrenergic receptors was shown to enhance the suppressive functions of T regulatory cells (Tregs) by enhancing CTLA-4 in a Protein Kinase A (PKA)-dependent pathway [48].

The presence of β_2_-adrenergic receptors on T cells is influenced by different factors, including environmental conditions, cytokines, genetic variations, and epigenetic processes such as histone acetylation. These factors can either increase or decrease the quantity of β_2_-adrenergic receptors, ultimately affecting the sensitivity and responsiveness of T cells to signals from the nervous system [41].

### Glucocorticoid receptor signaling in T cells

Glucocorticoids are also important players in the modification of T cell function. Glucocorticoids bind to cytoplasmic GC receptors to form an active complex with various effects. In the absence of GCs, GC receptors are found to be complexed with other proteins, such as heat shock proteins (HSPs), such as HSP70 and HSP90, and co-chaperone proteins. These keep the receptor inactive, stable, and help regulate receptor sensitivity and activation timing [49]. When GCs bind to the complex, the receptor-ligand complex is released and moves into the nucleus, allowing its interaction with a variety of transcription factors [50].

GCs can induce apoptosis in T cells, and sensitivity to this effect depends on the stage of T cell development [50]. The presence of certain signals, such as CD28-CD80/86, can protect T cells from GC-induced apoptosis. Similarly to β_2_-adrenergic receptors, the engagement of GC receptors in T cells has been observed to increase the population of highly immunosuppressive Tregs [50].

Importantly, these functional effects of GC signaling are closely linked to the metabolic state of T cells, as glucocorticoids are known to regulate genes involved in cellular metabolism and energy utilization. By modulating metabolic pathways, GCs can influence T cell differentiation, survival, and effector functions.

Different types of T cells rely on specific metabolic pathways for energy generation, with effector T cells utilizing glycolysis and other pathways, whereas memory and naïve T cells utilize oxidative phosphorylation and fatty acid oxidation. All these aspects can further influence T cell responses.

### Regulation of B cell responses during stress

B cells represent the major component of the adaptive humoral immune response and use their B cell receptors (BCR) to recognize antigens. Upon activation, B cells undergo clonal expansion and a proportion of B cells differentiate into plasma cells, which secrete large quantities of antibodies. Antibodies generated by plasma cells circulate through the body, bind to antigens, and form antigen-antibody complexes. These complexes elicit diverse immune responses, including direct pathogen neutralization, opsonization for phagocytosis by immune cells, and activation of the complement system to augment the immune response [51].

Currently, available evidence suggests a connection between psychological stress and impairment of the humoral immune response to immunization. In acute stress, stimulation of β_2_-adrenergic receptors suppresses Th1 immunity. At the same time, this effect enhances Th2 cytokines, and thus may support humoral immune responses [52]. High levels of chronic stress have been shown to be associated with a decrease in the concentration of protective antibodies against different pathogens, such as influenza, hepatitis B, and polysaccharides of Streptococcus pneumoniae [40]. One of the mechanisms underlying impaired humoral response is chronically increased cortisol levels.

Cortisol has been demonstrated to impair antibody production through multiple mechanisms. It can inhibit B cell proliferation by downregulating cyclins (e.g., cyclin D3) and upregulating cell cycle inhibitors such as p21 and p27, leading to cell cycle arrest in the G1 phase. In addition, cortisol suppresses key transcription factors, including X-box binding protein 1 (XBP-1) and B lymphocyte-induced maturation protein 1 (Blimp-1), which are essential for the differentiation of B cells into antibody-secreting plasma cells [53,54].

Cortisol also interferes with critical signaling pathways, such as NF-κB and activator protein 1 (AP-1), thereby impairing B cell activation. Furthermore, chronic exposure to cortisol promotes apoptosis by upregulating pro-apoptotic proteins and downregulating survival signals in lymphocytes. It also modulates cytokine production, reducing levels of B cell-supporting cytokines such as interleukin-2 (IL-2) and interleukin-21 (IL-21), while promoting an anti-inflammatory environment.

Collectively, these effects can disrupt germinal center formation, weaken long-term antibody responses, and ultimately result in reduced humoral immunity and diminished vaccine efficacy [53].

Several studies have examined the impact of stress on the immune system of students preparing for crucial oral academic examinations. In such cases, both elevated cortisol levels and suboptimal antibody responses have been observed in individuals experiencing academic stress [40].

In mouse models, an association has been observed between elevated glucocorticoid levels and a 50 % decline in B lymphocytes in the peripheral blood. This was attributed to the apoptosis of pre-B cells that emerged from the bone marrow [55].

In another study conducted among stressed graduate students, CD19^+^ B lymphocytes decreased significantly during the course of their first year of study. Interestingly, this study did not find a correlation between stress and CD3^+^ T lymphocytes [53]. There is a significant link between chronic stress and impaired secondary antibody responses to immunization, which is particularly evident among older adults [40]. In a comprehensive study conducted by Groer *et al*., the impact of prior experiences of sexual assault on the immune system was examined, with a specific focus on B-lymphocyte counts in female subjects. Interestingly, significant differences in B lymphocyte counts were evident between the analyzed groups, particularly highlighting that females with a history of sexual assault exhibited markedly diminished counts when compared to their unaffected counterparts [56].

### Phagocytic cells and their interaction with catecholamines during acute and chronic stress

As mentioned previously, activation of the sympathetic nervous system during acute stress induces rapid release of noradrenaline from sympathetic nerve endings, while adrenaline is concurrently secreted from the adrenal medulla into the circulation as a systemic hormone. Elevated levels of these catecholamines play a key role in modulating immune responses and affecting both adaptive and innate immune cells [31].

Phagocytic cells, particularly neutrophils and macrophages, are key targets of catecholaminergic signaling. Adrenaline and noradrenaline exert their effects predominantly through β_2_-adrenergic receptors. Their activation increases intracellular cyclic AMP (cAMP) and triggers downstream signaling pathways that suppress pro-inflammatory activity. Macrophages reduce the production of cytokines, such as TNF-α, IL-1, and IL-6, and increase secretion of the anti-inflammatory cytokine IL-10. Additionally, both neutrophils and macrophages exhibit decreased production of reactive oxygen species. This results in reduced microbicidal capacity. Phagocytosis may also be attenuated, although this effect is context-dependent [57].

Catecholamines also influence the migratory behavior of phagocytes. Changes in adhesion molecule expression and chemotactic responsiveness can limit the recruitment of phagocytes to sites of inflammation. Moreover, catecholaminergic signaling promotes polarization of macrophages toward the M2 phenotype, which is associated with anti-inflammatory functions and tissue repair rather than pathogen clearance. Collectively, these effects shift the immune response during acute stress toward a more regulatory and less cytotoxic profile [9].

Phagocytic cells are not merely passive recipients of neural signals. They are capable of synthesizing noradrenaline and express the full enzymatic machinery required for its production, including tyrosine hydroxylase, DOPA decarboxylase, and dopamine-β-hydroxylase. This capacity is inducible, particularly following activation by pathogen-associated molecular patterns (PAMPs), such as lipopolysaccharide (LPS) during bacterial infection. Noradrenaline produced by macrophages can be released into the extracellular space *via* vesicular exocytosis or membrane transporters and subsequently act in an autocrine or paracrine manner [58].

Macrophages also express enzymes responsible for catecholamine degradation, notably monoamine oxidase (MAO) and catechol-O-methyltransferase (COMT). These mechanisms enable tight local regulation of catecholamine concentrations, preventing excessive or prolonged signaling that could disrupt immune homeostasis or promote tissue damage [59].

Through their capacity for both synthesis and degradation of catecholamines, phagocytes function as autonomous regulatory units capable of shaping a localized neuroimmune microenvironment independently of direct neural input.

While acute catecholaminergic signaling *via* β_2_-adrenergic receptors generally exerts anti-inflammatory effects, this regulation is profoundly altered under conditions of chronic stress. Prolonged exposure to elevated catecholamine levels can lead to desensitization and downregulation of β-adrenergic receptors, resulting in diminished cAMP-mediated anti-inflammatory signaling. In parallel, chronic stress is associated with activation of pro-inflammatory transcriptional pathways, such as NF-κB, and the development of glucocorticoid resistance in immune cells. Consequently, rather than suppressing inflammation, sustained neuroendocrine activation promotes increased production of pro-inflammatory cytokines, including IL-6, TNF-α, and IL-1β. This shift reflects a transition from adaptive immunoregulation during acute stress to a maladaptive, low-grade inflammatory state under chronic stress conditions [60].

### Mucosal immunity under stress conditions

Mucosal immunity is a highly coordinated system that depends on several complementary mechanisms working together to protect vulnerable epithelial surfaces. Robust physical barriers, such as tightly joined epithelial cells and protective mucus layers, serve as the initial line of defense. In addition, the luminal environment contributes to immune protection through enzymatic digestion and chemical processing of potential antigens. Furthermore, mucosal immunity contains specific T cell subsets with specialized functions. All these aspects prevent the entry of pathogens and harmful substances [61].

Chronic stress has been demonstrated to profoundly influence the composition and function of the intestinal microbiota, as well as key aspects of immune regulation, including immune cell activity and cytokine production. Specifically, prolonged stress is associated with reduced microbial diversity and altered bacterial abundance, alongside dysregulation of immune cell populations [62]. Experimental evidence indicates that chronic stress disrupts the balance of immune cells, including intestinal mucosal immune (IMI) mast cells and CD4^+^ T lymphocytes, while simultaneously promoting increased production of pro-inflammatory cytokines such as IL-1, IL-6, and TNF-α [63].

Alterations in the gut microbiota further contribute to immune activation and this further triggers inflammatory responses and facilitates the development of conditions such as colitis. Notably, interventions targeting chronic stress have been shown to restore microbial homeostasis, modulate the tumor microenvironment, and significantly suppress tumor progression. Interestingly, selective serotonin reuptake inhibitors (SSRIs) appear to exert a protective effect in inflammatory bowel diseases, potentially by attenuating stress-induced inflammatory exacerbations within the intestinal mucosa [64].

### The effects of stress on immune progenitors

Interestingly, examining hematopoietic stem cells (HSCs) reveals that stress exerts upstream effects on immune function at the progenitor level. Chronic stress activates HSCs in the bone marrow, prompting their exit from quiescence and inducing proliferation. This process is often associated with an increased production of inflammatory cell subtypes, particularly monocytes and neutrophils. Mechanistically, this response appears to be mediated by sympathetic nervous system activity, specifically through the release of excess noradrenaline from sympathetic nerve fibers. Noradrenaline binds to β_3_-adrenergic receptors on bone marrow niche cells, leading to a reduction in CXCL12 expression. This decrease in CXCL12 disrupts the retention of HSCs within their niche, thereby promoting their proliferation and differentiation [65].

Thus, heightened HSC activity drives the increased generation of neutrophils and pro-inflammatory monocytes, and moreover, inflammatory signaling pathways also regulate hematopoiesis. This occurs across various contexts, including embryonic development, stress-induced (emergency) granulopoiesis, and age-related hematopoietic alterations. Collectively, these processes further underscore the link between chronic stress and enhanced inflammatory responses which is associated with skewed differentiation towards the myeloid lineage [66].

## Stress driven immune dysregulation

### Stress as a cause of secondary immunodeficiency

**Fig. 2:**
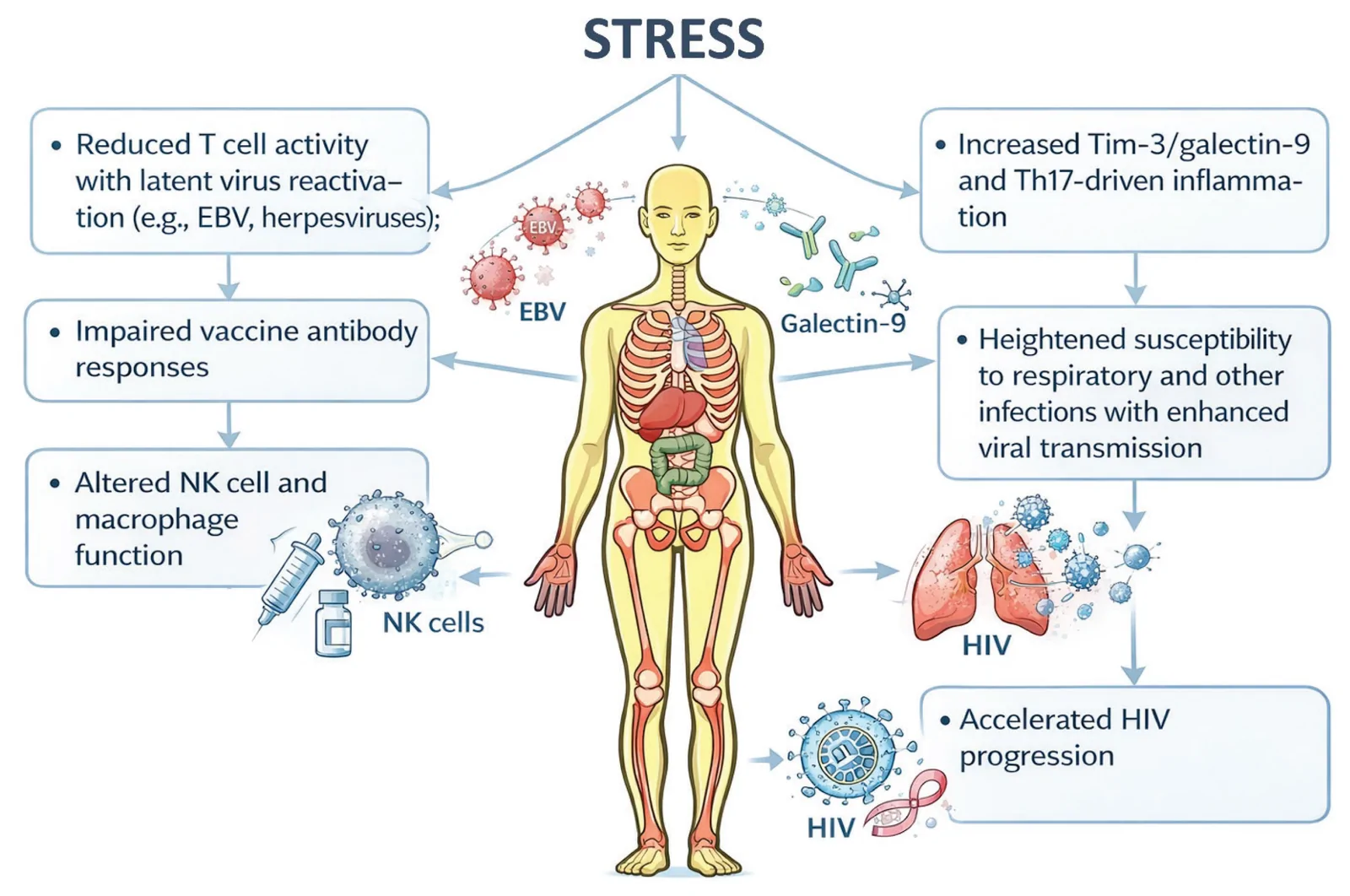
The multifaceted effects of stress on immunodeficiency. This diagram highlights the diverse effects of stress on immune system functions. Created at LeonardoAI.

It is well acknowledged that there are widespread consequences of stress on immune regulation across different domains of health (Fig. 2). Numerous studies have shown that stress can suppress the immune system in animals and humans. This suppression manifests through reduced antibody production, impaired cellular cytotoxicity, diminished phagocytic activity, and altered cytokine signaling across both innate and adaptive immune responses.

When illustrating how chronic stress exposure can lead to a state of functional immunodeficiency, studies most commonly report two distinct but related outcomes, increased susceptibility to infectious diseases and an attenuated immune response following vaccination. While increased susceptibility reflects a higher likelihood of developing infections, impaired vaccine responses indicate reduced ability to mount effective antigen-specific immunity.

Research conducted in mice subjected to cold water stress for several days revealed decreased T cell activity, reduced NK cell function, and altered macrophage physiology, characterized by increased prostaglandin E2 (PGE2) secretion, decreased MHC class II antigen expression, and elevated interleukin 1 (IL-1) levels [67].

In another study, the effect of chronic stress on immune function was observed in mice subjected to chronic physical restraint stress. This study showed that chronic stress increases the expression of checkpoint molecules, Tim-3 and galectin-9. Interestingly, the role of Tim-3 in chronic stress-induced immune suppression was shown to be important in regulating autophagy. Chronic stress caused autophagy in splenocytes through Tim-3, and importantly, by blocking Tim-3, excessive autophagy was reduced [68].

A recent study examined the effect of social disruption stress (SDR), induced by repeated social defeat over six nights, on immune responses to Mycobacterium tuberculosis in young and old mice. In older mice, SDR prior to infection promoted differentiation of IL-10-secreting T cells and impaired infection control [69].

Research in human populations, particularly among university students, who frequently report elevated levels of stress and anxiety, has shown that psychological stress is associated with increased susceptibility to illnesses such as influenza and the common cold [26,70].

In two different studies, researchers exposed study participants to two different types of rhinoviruses (RV-21 and 39). In the first study with 276 participants, it was demonstrated that when individuals experienced stressful events that lasted for a month or longer, their immune cells became less sensitive to cortisol. This reduced sensitivity to cortisol, in turn, has been linked to a higher risk of these individuals subsequently developing a cold [71]. In the second study involving 72 participants, it was shown that lower sensitivity to cortisol was associated with higher levels of proinflammatory cytokines after rhinovirus infection [71].

As the quality of the humoral immune response can be evaluated by the ability of plasma cells to produce specific antibodies, a meta-analysis of 13 studies evaluated the effect of stress on antibody responses to influenza virus vaccination. This meta-analysis showed that regardless of individual age, only 41 % of stressed individuals mounted sufficient antibody responses, while in non-stressed individuals, up to 59 % showed sufficient antibody responses [72].

In another study, it was observed that parents responsible for the care of children with developmental disabilities exhibited reduced antibody responses to the pneumococcal vaccine compared to appropriately matched control subjects. This effect was particularly evident among younger parents tasked with caring for their children with developmental disabilities [73].

Numerous studies have shown that stress is a major contributor to the reactivation of latent viruses. For instance, increased IgG antibody titers against Ebstein-Baar virus (EBV) viral capsid antigen (VCA) have been observed in the context of bereavement or divorce, exam stress, and perceived discrimination [74]. Human and animal studies indicate that stress can suppress antiviral T cell responses sufficiently to permit viral reactivation. In a mouse model, dominant animals experiencing more frequent social conflict showed higher rates of herpes simplex virus-1 reactivation, demonstrating a positive association between social stress and active infection [75].

A substantial body of evidence points to a relationship between chronic stress and the rate of HIV disease progression. A study by Garcia-Linares demonstrated that both physical and psychological abuse adversely affect health by increasing the likelihood of viral reactivation and impairing the body’s ability to suppress viral proliferation [76]. Furthermore, stress has been demonstrated to correlate with reduced CD4^+^ cell counts, elevated viral load, and accelerated disease progression in HIV patients [77].

### Stress as a trigger for autoimmune disorders

Autoimmune diseases are chronic disorders caused by loss of self-tolerance resulting from genetic and environmental factors, with stress acting as a potential trigger in predisposed individuals. Autoantibodies may appear years before clinical onset (preclinical autoimmunity), and stress-activated neuroendocrine pathways (HPA axis and sympathetic nervous system) increase proinflammatory cytokine production *via* NF-κB while altering T cell regulation, with cortisol providing partial negative feedback on immune activation. Additionally, stress-induced alterations in the microbiome may contribute significantly, potentially leading to an autoimmune shift *via* molecular mimicry [78,79].

Rheumatoid arthritis (RA) is the most thoroughly researched autoimmune disease with respect to the impact of stress. In a prospective cohort study conducted by Polinski *et al*., the Perceived Stress Scale-14 (PSS-14) was completed by participants who did not have inflammatory arthritis (IA) but were either first-degree relatives of individuals with RA or had tested positive for anti-cyclic citrullinated peptide autoantibodies (ACPA). The study found that participants with higher levels of perceived stress were at an increased risk of developing IA [80].

A study by Germain *et al*. demonstrated that female patients with RA more frequently attributed the onset of their symptoms to a life event than healthy controls. No significant difference was observed when comparing male RA patients with male controls, which suggests a potential gender difference in the perceived role of life events in the onset of RA symptoms [81].

Evers *et al*. demonstrated that worry predicted increased RA activity and pain in the following month and higher stress levels increased fatigue [82].

Similar to rheumatoid arthritis, stress significantly influences disease activity in systemic lupus erythematosus (SLE), an autoimmune disorder characterized by unpredictable flares, multi-organ damage and increased mortality [83–85].

A study by Patterson *et al*. found that individuals who experienced increased stress had significantly higher symptom burden and worse disease activity than those whose stress levels remained stable or declined [83].

In a study by Roberts *et al*., women who experienced trauma and had high levels of post-traumatic stress disorder (PTSD) symptoms had nearly a threefold increased risk of developing SLE [84]. Additionally, a study by Case *et al*. revealed that patients with a prior diagnosis of PTSD were twice as likely to receive a subsequent diagnosis of SLE [86].

In a study conducted by Feldman *et al*., which included a cohort of 67,516 women, exposure to physical and emotional abuse was associated with a 2.57-fold risk of developing SLE. Reported forms of abuse included severe physical punishment during childhood, harsh disciplinary practices, and exposure to verbal hostility or demeaning language [85]. Similarly, women who experienced sexual or physical abuse during childhood had a significantly increased risk of developing SLE in adulthood [87].

Multiple Sclerosis (MS) is a chronic autoimmune disease in which the immune system attacks the myelin sheath that protects the nerve fibers in the central nervous system. This leads to disrupted communication between the brain and the rest of the body, causing a wide range of symptoms such as muscle weakness, coordination, and vision difficulties [88–90].

Stress is thought to be a triggering factor for MS, with psychological stress possibly contributing to both the onset and relapse of the condition. More than four decades ago, Warren *et al*. conducted a comparative study revealing that a significantly higher proportion of patients with MS experienced unusual stress in the two-year period preceding the onset of the disease compared to controls. Furthermore, the study indicated that patients with MS endured a greater overall number of stressful life events [88].

A study conducted by Mohr *et al*. demonstrated a significant correlation between stressful life events and subsequent exacerbations in MS [89].

In a prospective longitudinal cohort study by Ackerman *et al*., interpersonal, work-related, financial, health, and other stressful events were similarly linked to MS exacerbations. Moreover, the frequency of life events was positively associated with the proportion of weeks of experiencing MS exacerbations [90].

Thyroid autoimmunity is also linked to stress. The majority of case-control studies have indicated that stress is a contributing factor influencing the onset and clinical progression of Graves’ disease [91]. Willems *et al*. reported 11 cases of stress-induced Graves’ disease following bereavement or illness of relatives in which stress relief alone led to remission without antithyroid therapy. Consistently, earlier studies show that adjunctive psychotherapy or benzodiazepines alongside antithyroid drugs promotes earlier remission and reduces recurrence rates [92].

Vita *et al*. conducted a 21-year longitudinal study on patients with Graves’ disease and reported that all patients who experienced exacerbations or relapses experienced at least one stressful event preceding each episode. Interestingly, the study showed that the younger the patient, the shorter the time lag between the stressful event and the onset of hyperthyroidism [93].

### The link between stress and allergy

Allergies represent a distinct group of diseases resulting from immune system dysfunction. These conditions arise due to an overreaction of the immune system to common and environmental antigens, presenting in various forms, from skin disorders such as atopic eczema to systemic reactions such as anaphylaxis [94]. Although stress is not considered a causative factor in the onset of allergies, it is well documented that it significantly amplifies the severity of allergic symptoms.

Atopic dermatitis (AD) is a distinct type of eczema and the most prevalent chronic inflammatory skin condition. In a recent study by Lönndahl, significant psychological triggers that exacerbated AD symptoms were identified. These include family issues, financial difficulties, work overload, school exam periods, lack of structure at work, and unforeseen events [95]. In this study, patients reported that psychological stress influenced both eczema and pruritus. Some noted that psychological stress preceded the onset of eczema, whereas others observed that psychological stress was a consequence of eczema, affecting its spread, duration, and severity [95].

A study by Wang *et al*. involving 19,381 mothers demonstrated that the children of mothers who worked during pregnancy had an increased risk of developing AD compared to those whose mothers did not work. Research revealed a dose-response relationship, in which the risk of AD increased proportionally with the level of maternal work stress during pregnancy [96]. Mitchel *et al*. investigated the relationships between parenting behaviors, skin care management, and disease severity in children with AD. The findings demonstrated that parents’ self-efficacy in managing both child behavior and AD was closely linked, with ineffective parenting strategies correlating with more severe AD [97].

In a prospective cohort study involving 4,898 children, McKenzie *et al*. identified a significant association between maternal depression in the postpartum period and prevalence of AD throughout childhood and adolescence. This study revealed that a history of postpartum depression was particularly linked to the occurrence of AD at ages 5 and 9 years. Furthermore, postpartum depression is associated with a higher likelihood of persistent AD [98].

Nash *et al*. conducted a longitudinal study investigating the link between domestic violence and the onset of multiple atopic diseases in adults using data from a large, representative United Kingdom (UK) database. This retrospective, population-based study examined 13,852 women who had experienced domestic violence and found a 52 % higher risk of developing atopic diseases, such as AD, asthma, and allergic rhinoconjunctivitis. The association was especially pronounced for asthma and allergic rhinoconjunctivitis compared to atopic eczema [99].

Asthma is a chronic inflammatory disorder of the airways frequently linked to allergic conditions and upper airway disease, and it commonly coexists with extra-respiratory atopic disorders such as food allergy and atopic dermatitis [100]. Its key features include persistent airway inflammation, variable airflow obstruction, airway hyperresponsiveness to specific and nonspecific stimuli, and structural alterations collectively known as airway remodeling [101,102].

Studies have demonstrated that stress amplifies Th2 and Th17 immune responses and weakens Treg cell responses, which are strongly linked to asthma exacerbation [103]. A meta-analysis by Wang *et al*. evaluated multiple studies and concluded that intimate partner violence (IPV) is associated with an increased incidence of childhood asthma [104]. Additionally, a study by Suglia *et al*. revealed striking results regarding the effect of positive caregiving practices, demonstrating that a mother’s ability to frequently engage in high levels of mother-child activities can significantly reduce the impact of violence on the risk of asthma in children [105].

A history of physical or sexual abuse has been associated with an approximately twofold increase in the odds of current asthma and asthma medication use [106].

Similarly, among residents of the New York area exposed to the World Trade Center disaster, individuals with probable PTSD exhibited a 1.65-fold increased risk of asthma diagnosis after 9/11, even after adjustment for sociodemographic, environmental, and lifestyle factors [107]. Consistent with these findings, research in rescue and recovery workers from the same event demonstrated that PTSD was associated with poorer asthma control and reduced quality of life [108].

In a cohort of more than 500 women, PTSD and depression severity were significantly higher in individuals with asthma compared to those without [109]. Likewise, data from a large, diverse, community-based sample in Dallas County revealed that asthma diagnosis was significantly associated with a history of mental health disorders and a 64 % increased likelihood of depressive symptoms, independent of age [110].

## Immune system dysfunction in response to specific stressors

### Immune changes after the death of a loved one: effects of grief

Losing someone close can cause emotional shattering. In particular, a child’s death is considered among the most profoundly traumatic life experiences, which is associated with a 32 % increase in the likelihood of early mortality among parents [111]. Numerous studies have reported alterations in the immune function associated with bereavement. These studies varied not only in the specific immunological variables measured, but also in the nature of the bereavement, which included the loss of a child, spouse, family member, or friend. Such differences in the relationship with the deceased may influence the intensity and type of grief experienced, potentially leading to variations in immune responses across studies.

In 1977, Bartrop *et al*. reported the findings of a prospective study involving twenty-six bereaved spouses. Although no significant differences were observed in T and B lymphocyte counts, protein levels, or the presence of autoantibodies, the bereaved group exhibited significant suppression of specific immune responses. This was particularly evident in the proliferative responses of lymphocytes to phytohemagglutinin and concavalin A, which are indicators of immune function [112].

Spratt and Denney measured immune system alterations in parents who experienced the sudden death of a previously healthy child. Their study revealed that bereaved parents exhibited a lower CD8 T cell count, a higher CD4 T cell count, and a shifted CD4:CD8 T cell ratio when compared to non-bereaved controls [113].

Irwin *et al*. demonstrated significantly reduced NK cell activity in widows and women experiencing anticipatory grief compared with non-bereaved controls [114].

Phillips *et al*. demonstrated a reduced antibody response to the influenza vaccine in individuals who had lost a spouse, relative, or close friend compared to responses of controls [115].

Similarly, Buckley *et al*. reported dynamic changes in immune markers after bereavement. Two weeks post-loss, they observed an elevated neutrophil count and increased platelet-granulocyte aggregates, reflecting heightened immune and inflammatory activity. However, at six months post-loss, there was a notable reduction in neutrophil, monocyte, and eosinophil counts, along with a decrease in platelet levels, suggesting a shift toward immune suppression over time as grief progresses [116].

Cankaya *et al*. reported significantly elevated levels of IL-6 in individuals who had experienced a sudden, unexpected loss of a close friend or loved one [117].

Lopez *et al*. reported that bereaved spouses who frequently used expressive suppression (conscious inhibition of emotional expression) showed increased inflammation, reflected by higher composite cytokine scores (IL-17A, IL-2, IL-6, TNF-α, IFN-γ). Individually, TNF-α and IFN-γ were significantly elevated, indicating that emotional suppression may amplify physiological stress-related systemic inflammatory activity [118].

In a prospective study conducted by Lillberg *et al*., data from a cohort of 10,808 women were analyzed to examine the relationship between significant life stressors and breast cancer risk. The findings indicated that the experience of losing a spouse or the death of a close relative or friend was associated with a heightened risk of developing breast cancer, highlighting the impact of psychological stressors, specifically those related to bereavement and loss, on physical health outcomes [119].

Table 2 shows a concise overview of selected studies that focused on specific stressors in correlation with immune functions.

### Intimate partner violence and immune response

Intimate partner violence (IPV) involves harmful actions directed towards a romantic partner, such as physical abuse, sexual assault, stalking, emotional abuse, and coercive control. Individuals subjected to IPV often suffer severe health consequences, in contrast to those who have not encountered such violence.

A study by Newton *et al*. examined inflammatory markers in 68 apparently healthy midlife women with a history of IPV. The researchers found that women with a history of stalking had significantly higher levels of C-reactive protein (CRP), an indicator of inflammation, although this association weakened after adjusting for BMI. Additionally, a history of physical assault was significantly associated with reduced IL-6 production in response to PHA stimulation, with the effect most pronounced in cases of severe assault [120].

Multiple studies support an association between IPV and inflammation. Blackmore *et al*. examined 171 pregnant women and measured serum pro-inflammatory cytokines. Seven (4.1 %) reported physical abuse in the past year (four during pregnancy); three felt unsafe in their current relationship and six due to a previous partner. Women with an IPV history showed significantly higher TNF-α levels (p<0.05) than non-exposed participants [121].

Garcia-Linares *et al*. reported that women exposed to physical abuse exhibited significantly reduced viral neutralization capacity compared with other groups, whereas psychologically abused women showed intermediate responses. Concordantly, HSV-1-specific antibody titers were lower in physically abused participants [76].

A study conducted by Groer *et al*. investigated the immune responses in women reporting a first-time rape. The comparison group consisted of healthy women who reported low stress levels, were medication-free, and, with one postmenopausal exception, were in the follicular phase of their menstrual cycles [56]. Flow cytometry showed immune alterations in rape survivors: higher CD8^+^ cells (10 % vs. 6.1 % controls) and lower CD19^+^ B cells (6.1 % vs. 19.8 %). Serum cortisol positively correlated with CD8^+^ counts. PHA-stimulated lymphocyte proliferation was reduced, indicating suppressed T cell function. Survivors also had elevated CRP, IL-10, and IL-6, most prominent 24–72 h post-assault, alongside decreased proliferation and a reduced Th1/Th2 ratio.

A different study by Romero-Martínez *et al*. examined IPV perpetrators undergoing the Trier Social Stress Test, involving public speaking and arithmetic tasks. The mental arithmetic tests are widely regarded as a standardized psychophysiological stressors of moderate intensity [122]. Salivary immunoglobulin A (sIgA) was measured as a mucosal immune marker. IPV perpetrators showed increased sIgA during the anticipatory phase prior to task performance, and within this group sIgA positively correlated with anger expression and testosterone. The findings suggest anger-related activation of mucosal immunity in IPV perpetrators [123].

### How child maltreatment influences immunity

Childhood maltreatment includes various forms of abuse and neglect such as emotional abuse, emotional neglect, and physical neglect. Emotional maltreatment, such as verbal abuse by parents or peers, can have severe consequences. Verbal abuse involves behaviors such as insults, humiliation, threats of abandonment or harm, and hurtful statements such as, “I wish you were never born”. These types of early life stress are among the most significant risk factors for developing various mental health disorders later in life [124]. Childhood maltreatment is an independent risk factor for inflammation in adulthood. A longitudinal prospective study by Danese *et al*. analyzed 1,037 participants and assessed childhood maltreatment (ages 3–11) across five adversities: maternal rejection, harsh discipline, caregiver disruption, physical abuse and sexual abuse (64 % none, 27 % single, 9 % multiple exposures). Maltreatment showed a graded association with adult inflammation approximately 20 years later, including elevated C-reactive protein, fibrinogen and leukocyte counts [125].

Bellis *et al*. conducted an exploratory study in 14 girls aged 8–15 years who had been sexually abused and underwent plasma antinuclear antibody (ANA) testing and thyroid function assessment. Although thyroid function was normal, the abused group showed a significantly higher prevalence of positive ANA titers than a control group of 22 healthy female volunteers. These findings suggest that sexually abused girls may exhibit disrupted immune homeostasis [126].

In a population-based Swedish cohort study conducted by Sepa *et al*., involving the first 5,986 consecutive children and their families with available 2.5-year follow-up data, it was found that maternal experiences of divorce and exposure to violence were significantly associated with the development of DM-related autoimmunity in children. This association persisted even after controlling for a family history of type 1 diabetes, indicating an independent link between these maternal stressors and autoimmune outcomes in the offspring [127]. Similarly, negative life events occurring within the first two years of life, particularly those involving challenging adaptations, child behavioral issues, and more chaotic family environments, have been associated with an increased risk of developing insulin-dependent diabetes mellitus (IDDM) [128].

A study of 15,357 adults (54 % women) examined childhood abuse (emotional, physical, sexual) and household dysfunction in relation to 21 autoimmune diseases categorized as Th1-driven, Th2-driven, rheumatic Th2, and mixed Th1/Th2. Thirty-seven percent reported ≥2 adverse childhood experiences (ACEs). Individuals with ≥2 ACEs had higher hospitalization risk: 70 % for Th1, 80 % for Th2, and 100 % for rheumatic disease. Risk of first autoimmune hospitalization increased with ACE number (p<0.05) [129].

Another study examined whether childhood bullying influences inflammation, measured by CRP levels, both short-term (ages 9–16) and in early adulthood (19–21). It followed 1,420 children and compared pure victims, pure bullies, bully-victims, and uninvolved peers. In the short term, bullied children showed a dose-dependent rise in CRP. Moreover, more frequent bullying was linked to higher CRP levels, suggesting a connection between bullying and low-grade inflammation [130].

Ayaydin *et al*. examined immune system alterations in adolescents who were victims of sexual abuse and developed PTSD. The sample included 33 patients (27 females, 6 males; ages 13–18) compared with controls. Among them, 18 experienced repeated abuse and 8 single-incident abuse; 11 also had physical abuse while 15 did not.

PTSD patients showed significantly reduced IFN-γ levels versus controls, especially after more severe trauma (repeated abuse or penetration). Reduced IFN-γ and lower CD3^+^ HLA-DR^+^ T-lymphocyte counts indicated impaired Th1/NK1 function and suppressed cellular immunity. Repeated-abuse victims had lower IFN-γ and CD3^+^ HLA-DR^+^ counts than single-incident victims [131].

Hartmann do Prado *et al*. studied 243 adolescents with early life stress (ELS) but no current psychopathology, assessing immune activation, regulatory lymphocytes, Th1/Th2/Th17 cytokines, and intracellular pathways (MAPKs p38/ERK, NF-κB). Reported stressors included sexual (25 %), physical (53.1 %), and emotional abuse (56.3 %), plus physical (56.3 %) and emotional neglect (37.5 %).

Adolescents with ELS showed immune dysregulation marked by an increase in activated T cells (CD3^+^CD4^+^CD25^+^, CD3^+^CD69^+^), regulatory T cells (CD8^+^CD28^−^, CD4^+^CD28^−^), reduced NK/NKT cells, elevated pro-inflammatory cytokines (IL-2, IL-4, IFN-γ, IL-17), and activation of MAPK and NF-κB pathways. These findings indicated persistent immune activation, possible early T cell senescence, and long-term physiological burden [132].

Furthermore, PTSD patients who endured both sexual and physical abuse demonstrated a trend towards reduced CD3^+^ and CD4^+^ T cell counts, with significantly lower total lymphocyte and CD19^+^ B-lymphocyte counts than those who were not physically abused. Additionally, adolescents who attempted suicide after experiencing childhood sexual trauma showed significantly elevated eosinophil counts and percentages. Overall, the study demonstrated pronounced immunosuppression in PTSD patients subjected to repeated abuse, physical abuse, and attempted suicide, reflecting a marked suppression of acquired immunity in these subgroups [131].

Bielas *et al*. examined lymphocyte subsets in maltreated children with depression or PTSD. Sixteen children (6–17 years), assessed 1–3 years after Child Protection Team intervention, were compared with 14 healthy controls.

Maltreated children with depression showed reduced recent thymic emigrants (RTEs) and T helper cells (age-adjusted), while PTSD was associated with fewer naïve T cells; RTE percentages were also lower than in controls. These findings indicate trauma-related alterations in T cell development that may influence long-term immune health [133].

### Connection between caregiving and immune functions

Numerous reports have indicated high levels of psychological distress among spouses caring for partners with dementia. Similarly, parents caring for children with disabilities experienced high levels of chronic stress [134].

Verdhara *et al*. studied 50 dementia spousal caregivers (median age 73) and 67 non-caregivers (median age 68) after trivalent influenza vaccination, measuring IgG on days 0, 7, 14, and 28. Only 16 % of caregivers versus 39 % of controls showed a four-fold antibody increase (p=0.007). Caregivers also had higher salivary cortisol, indicating greater HPA-axis activation and a weaker vaccine antibody response [135].

In a cross-sectional study conducted by Gouin *et al*., which included 53 caregivers and 77 non-caregiving controls, findings indicated that the cumulative effect of daily stressors contributed to elevations in inflammatory markers. Caregivers were more likely to experience multiple stressors within the past 24 h than non-caregiving controls. Moreover, the presence of multiple daily stressors was associated with significantly higher serum levels of inflammatory markers, including interleukin-6 (IL-6) and C-reactive protein (CRP), indicating that chronic exposure to daily stress may exacerbate inflammatory processes in caregivers [136].

In a study by Bauer *et al*., 49 spousal caregivers of dementia patients were examined, most caring for individuals with probable Alzheimer’s disease (78 %), followed by multi-infarct dementia (17 %) and Parkinson’s disease with progressive dementia (5 %). Caregivers were compared with non-caregivers across psychological and physiological outcomes, with particular attention to immune parameters. Caregivers reported significantly higher anxiety, depression, and stress levels, accompanied by elevated salivary cortisol, indicating chronic activation of the HPA axis. Although full blood counts did not differ between groups, marked differences emerged in cellular immune function. Caregivers showed significantly reduced PHA-stimulated lymphocyte proliferation, demonstrating impaired T cell responsiveness. Furthermore, while basal IL-2 production remained similar, stimulated IL-2 production was approximately 47 % lower in caregivers. These findings suggest that chronic caregiving stress suppresses adaptive immune activity, particularly T cell activation and cytokine signaling [137].

Another study involving 121 elderly Alzheimer’s caregivers over four years demonstrated that reduced satisfaction with leisure activities was associated with elevated levels of specific inflammatory markers, namely TNF-α, IL-8, and interferon-gamma. The findings suggest that for Alzheimer’s caregivers, engaging in leisure activities might play a role in modulating inflammation, potentially impacting their overall well-being and mental health [138].

Another study explored the connection between long-term stress and IL-6 levels in 119 men and women caring for a spouse with dementia and 106 people who were not caregivers. Researchers measured IL-6 levels and related health habits over a six-year period. In this study, it was shown that caregivers’ IL-6 levels increased at an average rate approximately four times higher than that of non-caregivers. Interestingly, former caregivers showed similar increases in IL-6 levels as current caregivers, even several years after their spouses passed away [139].

Vitlic *et al*. investigated innate immune competence by assessing neutrophil function in two caregiver populations: younger parents of children with developmental disabilities and older adults providing full-time care for spouses with dementia. Overall neutrophil activity was preserved across caregivers; however, individuals reporting the highest psychological distress exhibited significantly reduced neutrophil phagocytic capacity. These findings indicate that caregiving stress does not uniformly impair innate immunity but selectively compromises key antimicrobial effector functions under conditions of high psychological burden [140].

### Immune dysregulation in PTSD and trauma survivors

PTSD is a health condition that develops after an individual is exposed to a traumatic event. It involves symptoms such as intrusive memories, efforts to avoid reminders of trauma, shifts in mood and thinking, and heightened levels of arousal and responsiveness [141–143].

Research has long explored the immune system changes associated with PTSD, with most findings indicating significant effects on immune function in patients with the condition. Numerous studies have shown a connection between PTSD and accelerated immune aging. Aiello *et al*. conducted a study with 85 PTSD-affected participants, revealing that recent PTSD (within the past year) was correlated with notable shifts in the CD8^+^ T cell population. These include an increased ratio of late-differentiated effector T cells to naïve T cells, higher proportions of KLRG1^+^ and CD57^+^ cells, and a significantly elevated percentage of CD57^+^ cells in the CD4 subset. Additionally, individuals with recent PTSD had a lower CD4 ratio. Lifetime PTSD has been linked to variations in multiple indicators of immune aging [141].

Similarly, Sommershof *et al*. examined 19 individuals with PTSD related to war and torture and compared them with 27 non-PTSD controls. The results revealed a 32 % reduction (p=0.01) in the proportion of naïve CD8^+^ T lymphocytes in patients with PTSD. Conversely, the proportions of CD3^+^ central memory T lymphocytes and effector memory T lymphocytes were significantly increased by 22 % and 34 %, respectively, in individuals with PTSD compared to those in the control group. This effect was also observed in trauma-exposed individuals without PTSD, suggesting a cumulative impact of traumatic stress on T cell distribution [142].

Kosor *et al*. explored the impact of psychological stress on B-cell responses by examining the immune reaction to influenza vaccination in 28 patients with combat-related PTSD. This study revealed that patients with PTSD had a lower baseline count of influenza-specific CD8^+^ T cells prior to vaccination. However, no significant differences have been observed in the humoral or cellular immune responses generated after vaccination between patients with PTSD and controls [143].

A study of 62 war veterans assessed relative telomere length and basal telomerase activity in PBMCs from individuals with PTSD, age-matched controls, and elderly volunteers, alongside T cell proliferation, cytokine production, and immune checkpoint expression. Middle-aged veterans with PTSD had shorter telomeres than controls, although longer than the elderly group. PTSD was associated with impaired CD8^+^ T cell proliferation at high mitogen concentrations and reduced spontaneous IL-2 and IFN-γ production. In contrast, elderly participants showed broader immune alterations, including increased TNF-α and IL-6 and reduced overall T cell proliferation. Telomerase activity did not differ between PTSD and control groups. These findings indicate early immunosenescence in PTSD [144].

Pace *et al*. investigated the association between molecular signaling pathways involved in inflammation and childhood abuse-related PTSD in women, with a particular focus on the NF-κB pathway activity, a key regulator of inflammatory processes. Their findings revealed that women with PTSD exhibited significantly higher NF-κB activity than healthy controls. Furthermore, NF-κB activity was positively correlated with PTSD symptom severity [145].

A study by Ironson *et al*. focused on individuals living in neighborhoods severely impacted by Hurricane Andrew, evaluated between one and four months post-disaster. In this study, WBC counts were significantly elevated in individuals with greater degrees of loss and PTSD. NK cell cytotoxicity (NKCC) was lower among those affected, especially in individuals reporting increased somatic symptoms after a hurricane. Moreover, the community sample exhibited lower levels of NKCC, CD4, and CD8 cells, suggesting an overall suppression of immune function in these T cell subsets [146].

Similarly, Gotovac *et al*. conducted a study on Croatian war veterans with chronic combat-related PTSD, categorizing participants into two subgroups: compensation-seeking (n=15) and retired or non-compensation-seeking individuals (n=14). Both PTSD groups exhibited NKCC compared to the controls. This reduction in NKCC was not linked to perforin expression, as perforin levels were not lower than those in controls. Interestingly, elevated levels of perforin were observed in NK cells of retired PTSD subjects. Both groups showed increased relative expression of glucocorticoid receptors (GCR) in lymphocytes, while no significant difference in baseline serum cortisol levels was found between the subgroups [147].

In contrast, a study involving 10 Vietnam war combat veterans with long-term PTSD and 9 controls with comparable combat exposure and histories of alcohol abuse found no significant differences in hormone levels or lymphocyte phenotypes between the groups. However, patients with PTSD demonstrated elevated NK cell activity compared to controls [148].

## Discussion

Accumulating evidence demonstrates that psychological stress is a major regulator of immune system. Depending on its duration and intensity, stress can enhance or suppress immune activity and thereby contribute to immunodeficiency, autoimmunity, chronic inflammatory disorders, and cancer. These effects arise through alterations in cytokine profiles, intracellular signaling pathways, inflammatory mediator activation, cytotoxic lymphocyte function, phagocytic capacity, and antibody production.

During acute stress, hypothalamic activation triggers the sympathetic-adreno-medullary system to release adrenaline, while the HPA axis independently induces cortisol secretion *via* CRH-ACTH signaling. These hormones act on receptors expressed by immune cells, promoting leukocyte redistribution and increased trafficking, particularly of neutrophils, to sites of potential injury or infection.

In contrast, chronic stress disrupts immune regulation. Prolonged cortisol exposure leads to glucocorticoid receptor desensitization and impaired HPA feedback, resulting in dysregulated cytokine production, reduced antibody responses, and diminished immune cell function, ultimately weakening host defense.

Chronic stress impairs adaptive immunity by reducing T cell numbers, proliferation, and effector function while altering cytokine balance. Catecholamine signaling through β_2_-adrenergic receptors promotes IL-10 production, suppresses TNF-α and IFN-γ, disrupts T cell trafficking, and expands regulatory T cells. Activation of glucocorticoid receptors further inhibits T cell activation and IL-2 signaling, promotes apoptosis of activated T cells, and skews responses toward regulatory and Th2 phenotypes. In B cells, chronic stress lowers protective antibody production, increasing susceptibility to infection and reducing vaccine responsiveness.

Stress plays a key role in autoimmunity, with studies linking it to the onset and exacerbation of diseases, such as RA and systemic lupus erythematosus (SLE). Trauma and stress have been shown to increase the risk of developing autoimmune diseases, potentially through stress-induced inflammatory processes and microbiome alterations, which may trigger autoimmunity through molecular mimicry. Psychological stress has also been linked to allergic diseases such as asthma and AD. It has been shown that stress often precedes the onset of eczema, and parenting behaviors or domestic violence can exacerbate disease severity in children. Specific stressors, such as IPV or childhood maltreatment, are associated with an increased risk of atopic conditions, such as asthma, AD, and allergic rhinoconjunctivitis.

Specific serious life events, such as the death of a loved one or IPV, profoundly affect the immune cells. For example, CD8^+^ T cell responses are diminished in parents grieving the sudden death of a child, and NKCC is reduced in widows or individuals experiencing anticipatory grief. These stressors are associated with a heightened risk of illness, including cancer. Studies also suggest that IPV survivors experience inflammation and T cell suppression, whereas perpetrators of IPV may paradoxically experience improved immune status. Childhood maltreatment is an independent risk factor for inflammation in adulthood and is significantly associated with the development of autoimmune diseases and allergies. In spousal caregivers of patients with dementia, stress has been linked to weakened antibody response to vaccines.

This study has several limitations. One limitation of this study is the potential overlap of diagnoses, particularly PTSD, which is also prevalent among individuals with childhood maltreatment, IPV, or bereaved spouses. In the search for specific stressors, I primarily focused on studies evaluating broader categories of immune disorders such as immunodeficiencies or autoimmunity. Because specific stressors represent only a subsection of the entire manuscript, a comprehensive search combining individual stressors, such as childhood maltreatment, with every specific autoimmune condition (e.g., Hashimoto’s thyroiditis or Sjögren’s syndrome) has not been conducted. Consequently, the section on specific stressors did not include all existing studies published on this topic.

There are also several limitations to the reviewed studies. A major issue is that the intensity of stress is frequently assessed using self-report measures, which are subject to bias and limited comparability across studies. Furthermore, although stress is associated with immune alterations, other factors may be involved; therefore, establishing causality remains challenging.

## Conclusions

Considering the significant impact of stress on immune cells, manifesting as vaccine failures, prolonged inflammation, increased susceptibility to infections, onset and exacerbation of autoimmune conditions, and triggering of allergic responses, it is crucial to integrate insights from psychoneuroimmunology into multidisciplinary teams managing chronic illnesses and malignancies. For patients with a history of trauma or abuse, it is essential to address not only their heightened risk of mental health disorders, but also their vulnerability to immune-related diseases.

## Figures and Tables

**Table 1:** A summary table outlining key receptors expressed on major immune cell subsets, including their relative expression levels.

*Immune subset*	β1-AR	β2-AR	β3-AR	Likely stress sensitivity
*NK cells*	Low	High	Low/uncertain	Very high
*Monocytes (especially non-classical)*	Low	High	Low/uncertain	High
*Macrophages*	Low/inducible	Intermediate-high	Low/uncertain	High
*B cells*	Low	Intermediate-high	Low/uncertain	Moderate-high
*CD8 T cells (overall)*	Low	Intermediate	Very low/not detected in most settings	Moderate-high
*CD8 effector-memory T cells*	Low	High	Very low	High
*Exhausted CD8 T cells*	High	Low-intermediate	Not detected	High in chronic stress/chronic antigen settings
*CD4 T cells (bulk)*	Low	Low	Low/uncertain	Lower than CD8/NK/monocytes
*Th1 cells*	Low	Intermediate	Low/uncertain	Moderate
*Th2 cells*	Low	Absent/very low	Low/uncertain	Low
*Treg cells*	Low/uncertain	Intermediate	Low/uncertain	Moderate
*Dendritic cells*	Low	Intermediate	Low/uncertain	Moderate
*Neutrophils/granulocytes*	Low	Intermediate-high	Low/uncertain	Moderate–high
*MDSCs*	Low	Intermediate-high	Low/uncertain	High in cancer/chronic stress

**Table 2:** Overview of selected studies focusing on specific stressors in correlation with immune functions.

Author (year)	Findings	Journal
Bartrop *et al*. (1977)	Bereaved individuals exhibited significant suppression of specific immune responses, such as reduced T-cell activity.	Psychosomatic Medicine
Spratt and Denney (1991)	Parents who experienced the sudden death of a child showed lower CD8 T cell counts, higher CD4 T cell counts, and altered CD4:CD8 ratios.	Journal of the American Academy of Child & Adolescent Psychiatry
Irwin *et al*. (1987)	Widows and women experiencing anticipatory grief exhibited significantly reduced natural killer (NK) cell activity compared to controls.	Psychosomatic Medicine
Phillips *et al*. (2006)	Bereaved individuals showed a reduced antibody response to the influenza vaccine compared to controls.	Brain, Behavior, and Immunity
Buckley *et al*. (2012)	Post-bereavement, immune markers shifted from heightened activity to suppression over time, including changes in neutrophil counts.	Brain, Behavior, and Immunity
Cankaya *et al*. (2012)	Individuals experiencing sudden loss exhibited significantly elevated IL-6 levels, suggesting inflammation.	Psychiatry Research
Lopez *et al*. (2013)	Bereaved spouses who used expressive suppression exhibited heightened inflammatory responses, such as increased cytokine levels.	Psychosomatic Medicine
Lillberg *et al*. (2003)	Stressful life events, such as bereavement, were linked to an increased risk of breast cancer.	Cancer Causes & Control
Newton *et al*. (2011)	IPV survivors exhibited elevated CRP and altered IL-6 responses, indicating inflammation.	Brain, Behavior, and Immunity
Robertson Blackmore *et al*. (2011)	Women with a history of IPV had significantly elevated TNF-α levels during pregnancy.	American Journal of Obstetrics and Gynecology
Garcia-Linares *et al*. (2004)	Women experiencing physical abuse had the lowest virus neutralization levels and HSV-1-specific antibody titers compared to others.	Aggressive Behavior
Groer *et al*. (2010)	Rape survivors exhibited increased CD8 cytotoxic cells and reduced CD19 B cells compared to controls.	Biological Research for Nursing
Romero-Martínez *et al*. (2014)	IPV perpetrators showed higher salivary immunoglobulin A (sIgA) levels during stress tests, associated with anger expression.	Biological Psychology
Danese *et al*. (2007)	Childhood maltreatment predicted elevated CRP levels and increased inflammation in adulthood.	Proceedings of the National Academy of Sciences
Bellis *et al*. (1996)	Sexually abused girls exhibited higher antinuclear antibody (ANA) titers, indicating immune dysregulation.	Journal of Clinical Pathology
Sepa *et al*. (2005)	Maternal experiences of divorce and violence were significantly linked to diabetes-related autoimmunity in children.	Diabetologia
Dube *et al*. (2009)	Individuals with ≥2 adverse childhood experiences (ACEs) had significantly higher risks of autoimmune diseases.	Psychosomatic Medicine
Copeland *et al*. (2014)	Bullied children exhibited a dose-dependent increase in CRP levels, linking bullying to low-grade inflammation.	Proceedings of the National Academy of Sciences
Ayaydin *et al*. (2016)	Adolescents with PTSD due to sexual abuse showed reduced IFN-γ levels and suppressed cellular immunity.	Psychiatry Research
Hartmann do Prado *et al*. (2016)	Adolescents with early life stress exhibited increased activated T cells and elevated pro-inflammatory cytokines.	Neuropsychopharmacology

## Abbreviations

ACEs: Adverse childhood experiences
ACTH: Adrenocorticotropic hormone
AD: Atopic dermatitis
ATP: Adenosine triphosphate
BCR: B cell receptor
β_2_-adrenergic receptor: Beta-2 adrenergic receptor
cAMP: Cyclic adenosine monophosphate
CD3^+^: Cluster of differentiation 3
CD4^+^: Cluster of differentiation 4
CD8^+^: Cluster of differentiation 8
CD19^+^: Cluster of differentiation 19
CNS: Central nervous system
COMT: Catechol-O-methyltransferase
CRH: Corticotropin-releasing hormone
CRP: C-reactive protein
CTLA-4: Cytotoxic T-lymphocyte-associated protein 4
EBV: Epstein–Barr virus
ELS: Early life stress
GABA: Gamma-aminobutyric acid
GC receptor (GCR): Glucocorticoid receptor
GLUT1: Glucose transporter 1
HIF-1α: Hypoxia-inducible factor 1-alpha
HIV: Human immunodeficiency virus
HPA axis: Hypothalamic-Pituitary-Adrenal axis
HSV-1: Herpes simplex virus type 1
IFN-α: Interferon alpha
IFN-γ: Interferon gamma
IL-1: Interleukin 1
IL-2: Interleukin 2
IL-4: Interleukin 4
IL-6: Interleukin 6
IL-8: Interleukin 8
IL-10: Interleukin 10
IL-17A: Interleukin 17A
IPV: Intimate partner violence
LPS: Lipopolysaccharide
MAO: Monoamine oxidase
MAPK: Mitogen-activated protein kinase
MS: Multiple sclerosis
NF-κB: Nuclear factor kappa B
NK: Natural killer (cells)
NKCC: Natural killer cell cytotoxicity
ORCID: Open Researcher and Contributor ID
PAMPs: Pathogen-associated molecular patterns
PBMCs: Peripheral blood mononuclear cells
PD-1: Programmed cell death protein 1
PGE2: Prostaglandin E2
PKA: Protein kinase A
PNI: Psychoneuroimmunology
PTSD: Post-traumatic stress disorder
RA: Rheumatoid arthritis
RV-21/RV-39: Rhinovirus strains
SDR: Social disruption stress
sIgA: Salivary immunoglobulin A
SLE: Systemic lupus erythematosus
Th1/Th2/Th17: T helper cell subsets
Treg: Regulatory T cells
